# Validation of UPLC-MS/MS Method for Determination of Urinary Lactulose/Mannitol

**DOI:** 10.3390/molecules23102705

**Published:** 2018-10-20

**Authors:** Jacopo Gervasoni, Aniello Primiano, Cristina Graziani, Franco Scaldaferri, Antonio Gasbarrini, Andrea Urbani, Silvia Persichilli

**Affiliations:** 1 Fondazione Policlinico Universitario A. Gemelli IRCCS, 00168 Roma, Italy; graziani.cristina@gmail.com (C.G.); francoscaldaferri@gmail.com (F.S.); antonio.gasbarrini@unicatt.it (A.G.); andrea.urbani@policlinicogemelli.it (A.U.); silvia.persichilli@policlinicogemelli.it (S.P.); 2 Istituto di Biochimica e Biochimica Clinica, Università Cattolica del Sacro Cuore, 00168 Roma, Italy; aniello.primiano@unicatt.it; 3 Istituto di Patologia Speciale Medica e Semeiotica Medica, Università Cattolica del Sacro Cuore, 00168 Roma, Italy

**Keywords:** UPLC-MS/MS, intestinal permeability, L/M

## Abstract

Determination of urinary lactulose/mannitol is one of the most used tests to evaluate intestinal barrier function. High-performance liquid chromatography (HPLC) separation with electrospray ionization tandem mass spectrometry guarantees high levels of selectivity and reproducibility. In this paper we report an upgrade of the previous published liquid chromatography tandem mass spectrometry method, introducing more reliable internal standards and ultra-performance liquid chromatography with ethylene bridged hybrid amide columns. The ultra-performance liquid chromatography provided an efficient chromatographic separation of the two sugars in 5 min, compared to 15 min using the previous method. The limit of quantification was 10 µg/mL for mannitol and 2.5 µg/mL for lactulose, and the assay was linear up to 1000 µg/mL for mannitol and 1000 µg/mL for lactulose. The within-run precision and accuracy ranged from 0.7 to 2.9% and 97.2 to 101.2%, respectively. The between-run precision and accuracy ranged from 1.9 to 4.7% and 94.8 to 97.5%, respectively. Recovery was higher than 90.2% for both lactulose and mannitol, and the matrix effect for both lactulose and mannitol was lower than 15%. With this new method we have a real improvement in terms of accuracy and reproducibility, ensuring results in shorter time. The changes to the previous protocol make this method excellent for routine purposes.

## 1. Introduction

The lactulose/mannitol (L/M) test has been the most widespread dual-sugar test used to assess the intestinal barrier function over the last thirty years [1]. The absorption of the sugar alcohol mannitol and the disaccharide lactulose is directly related to transcellular and paracellular permeability, respectively, such that damage to mucosal cells and to tight junctions are associated with decreased mannitol and increased lactulose absorption [2,3].

There are variety of techniques used to determine lactulose, mannitol and sucrose in urine samples including enzymatic assay, gas-liquid chromatography coupled to a flame ionization detector (GC-FID), and high-performance liquid chromatography (HPLC) with refractive index detector [4,5,6]. Among these, a valid solution for carbohydrate analysis is HPLC separation with electrospray ionization tandem mass spectrometry (ESI-MS/MS), because of its high level of selectivity and reproducibility [7,8].

The published chromatographic methods for L/M analysis report the use of HILIC- or NH_2_-based columns, which adequately retain both lactulose and mannitol. Data from literature suggest that the estimation of the concentration of lactulose and mannitol is not always accurate, and there are no international references that facilitate the standardization of the lactulose and mannitol assay available [1]. For these reasons, the cut-off is generally defined by a single study or by a single laboratory [9].

We have recently published a validation of the HPLC-MS/MS method for lactulose and mannitol quantification using a NH_2_-column and a triple quadrupole mass spectrometry (MS) system with ESI-ionization. In this method, as according to other published methods, we used raffinose as the internal standard both for lactulose and mannitol [10]. 

As suggested by international guidelines, the use of labeled internal standards guarantees the highest reliability in terms of reproducibility and accuracy [11,12,13]. Aiming to introduce the lactulose/mannitol ratio determination in a high throughput routine clinical laboratory, we updated our published method by improving the chromatographic method (5 min vs. 15 min), and introduced an isotopic based internal standard calibration. Moreover, LC-MS/MS analysis was performed using a more sensitive instrument.

## 2. Results

**Figure 1:**
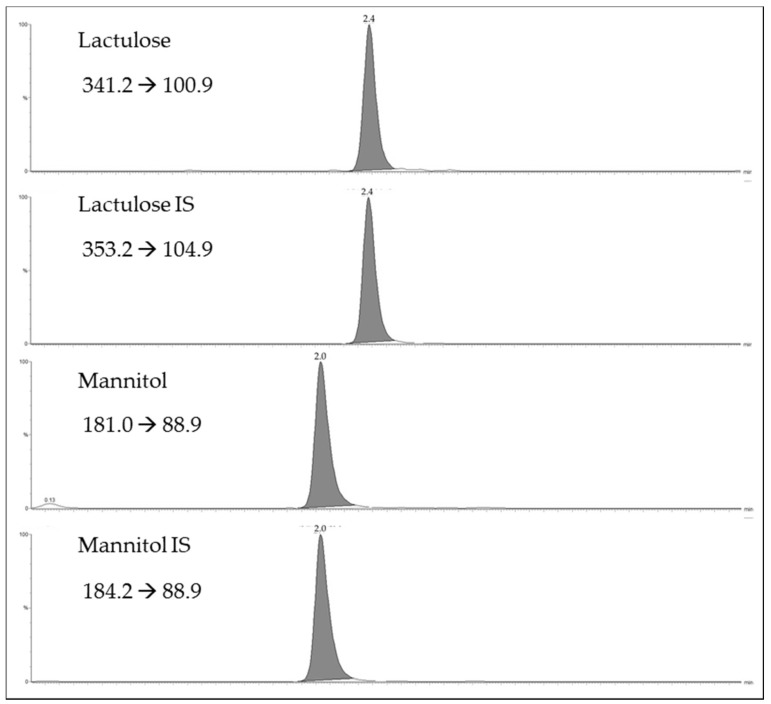
Selected reaction monitoring (SRM) chromatograms for lactulose, mannitol and their internal standards lactulose ^13^C_12_ and d-mannitol-1 ^13^C,1-1-d_2._

Figure 1 shows the selected reaction monitoring (SRM) chromatograms for the lactulose and mannitol, and their internal standards (IS) d-mannitol-1 ^13^C,1-1-d_2_ and lactulose ^13^C_12_ for a urinary sample. As shown in Figure 1, the ultra performance liquid chromatography (UPLC) provides efficient chromatographic separation of the two sugars in 5 min. The limits of detection were 2 µg/mL for mannitol and 0.5 µg/mL for lactulose. The limits of quantification were 10 µg/mL for mannitol and 2.5 µg/mL for lactulose. The assay was linear up to 1000 µg/mL for mannitol and up to 1000 µg/mL for lactulose. Eight calibration curves, analyzed over a period of three weeks, displayed an R^2^ always higher than 0.99 for both analytes. The slopes and intercepts of calibration curves prepared in solvent overlapped with those prepared in urinary matrix. (*Y*_mannitol/d-mannitol-_^13^_C,1-1-d2_ = −0.008 + 0.014*X*_nominal concentration_, *r* = 0.999, *Y*_mannitol/ d-mannitol-_^13^_C,1-1-d2_ = −0.039 + 0.013*X*_nominal concentration_, *r* = 0.999, respectively and *Y*_lactulose/lactulose_^13^_C12_ = −0.007 + 0.019*X*_nominal concentration_, *r* = 0.999, *Y*_lactulose/lactulose_^13^_C12_ = −0.029 + 0.018*X*_nominal concentration_, *r* = 0.999, respectively).

The precision and accuracy of the results are summarized in Table 1. The within-run precision and accuracy ranged from 0.7 to 2.9% and 97.2 to 101.2%, respectively. The between-run precision and accuracy ranged from 1.9 to 4.7% and 94.8 to 97.5%, respectively. Recovery was always higher than 90.2% for both lactulose and mannitol.

The matrix effect for both lactulose and mannitol was lower than 15%. 

The correlation analysis performed on 100 urinary samples showed a good agreement (R^2^ = 0.935) between this and previous method.

## 3. Discussion and Conclusions

L/M determination is a useful clinical test for assessing alterations in gut permeability. Among the different methods proposed for sugar analysis, the chromatographic ones are undoubtedly the most specific and sensitive.

Several studies reported the validation of HPLC methods for lactulose and mannitol using different detectors, but the lack of any international standard protocol makes it difficult to compare the results of different laboratories [9]. In addition to the pre analytical limitations, due to differences in the protocols such as dosage of sugar or collection time, several analytical findings can affect urine sugar determination. Therefore, the L/M cutoffs value should be accurately evaluated before being introduced to the clinical laboratory.

In order to improve the performance of our previously published method and to introduce it into the routine clinical laboratory setting, we developed and validated a more sensitive, rapid and robust method for the quantification of lactulose and mannitol in urine samples. In the previous paper we validated the HPLC-MS/MS method for lactulose and mannitol in urine using a silica-based amino stationary phase with a sample dilution procedure. Moreover, according to literature data, raffinose was used as the internal standard [10]. The use of silica-based amino phases were one of the best choices for sugar retention, taking advantage of the ion exchange interaction. However, the reduced form of sugars can interact with the amine residues of the stationary phase and decrease the column’s lifetime [14,15,16]. 

In this work we used a UPLC system equipped with a BEH amide column, a stationary phase that utilizes an amide phase, trifunctionally bonded to an ethylene bridged hybrid particle which operates in a wide range of pH, to ensure the requested retention of polar molecules through the hydrophilic interaction chromatography and to increase the column lifetime with respect to the amino phase [17]. In fact, the analysis of 200 urinary samples performed over a six-month period showed a stability of analytical performance in terms of retention time, peak shape and peak area, which were superior to the previous used NH_2_-column. Furthermore, using a better performing UPLC system run time, reduced from 15 to 5 min, allowed the analysis of a large number of samples over a period of time more suitable for a large routine laboratory.

Another further improvement of the presented method with respect to the previous one was the use of labeled analytes as internal standard instead of raffinose. Raffinose is a trisaccharide that displays different retention times of lactulose and mannitol and thus it does not guarantee the efficient correction of the matrix effect, but even so the use of this molecule is still widespread. The use of a labeled internal standard, which shows identical chemical properties of the analyte, should overcome the analytical problems such as stability, recovery, and ion suppression.

Only one paper reported the analysis of L/M by different HPLC and LC-MS/MS platforms and only two of them used a labeled internal standard. To our best knowledge, our paper first reported the complete validation of a LC-MS/MS method for L/M analysis using lactulose ^13^C_12_ and d-Mannitol-1 ^13^C,1-1-d_2_ as internal standard [14].

In conclusion, all these changes have allowed real improvements in terms of accuracy, precision and reproducibility, making this method great for routine purpose.

## 4. Materials and Methods

### 4.1. Chemicals and Reagents

Water and acetonitrile (LC-MS grade) were purchased from Merck (Merck KGaA, Darmstadt, German). Formic acid (98%, LC-MS grade) was purchased from Baker (Mallinckrodt Baker Italia, Milano, Italia) and d-Mannitol-1 ^13^C,1-1-d_2_ and lactulose ^13^C_12_ and ammonium formate were purchased from Sigma-Aldrich (Merck KGaA, Darmstadt, German).

Lactulose, mannitol and chlorhexidine were purchased from BioChemica (AppliChem GmbH, Darmstadt, Germany).

Stock solutions of mannitol (4 g/L) and lactulose (4 g/L) were prepared in water and stored at −80 °C. Internal standards stock solutions containing 500 μg/mL mannitol ^13^C_6_ and lactulose ^13^C_12_ were prepared in water and stored at −80 °C

Working solutions were prepared in water/acetonitrile (20/80, *v*/*v*) at concentrations of 1600 µg/mL for mannitol and 640 µg/mL for lactulose. Serial dilutions from working solutions were used to prepare seven-point calibration curves for both mannitol and lactulose (10-40-80-160-320-640 µg/mL; 2.5-10-20-40-80-160 µg/mL, respectively) and were kept at −20 °C until use. The calibration curve included a zero (only solvent) and a blank (solvent plus IS), which were not used for the construction of calibration curve. 

d-Mannitol-1 ^13^C,1-1-d_2_ and lactulose ^13^C_12_ stock solutions were diluted with acetonitrile to achieve a final concentration of 2.5 µg/mL and 5 µg/mL for lactulose ^13^C_12_ and d-Mannitol-1 ^13^C,1-1-d_2_, respectively.

### 4.2. Sample Collection and Treatment

Urine samples before the analysis for L/M ratio were left to thaw at room temperature, then stirred for 1 min using a Vortex mixer and then centrifuged at 5000 g for 4 min to remove the sediment according to the laboratory procedure.

For 10 μL of urine samples, controls and standards were added: 240 μL of IS solution and, after mixed, a 200 μL aliquot was transferred into a glass vial for the injection to HPLC-MS/MS.

### 4.3. Instrumentation

The LC-MS/MS system consisted of an Acquity UPLC system interfaced with a triple quadrupole mass spectrometer (Xevo TQS-Micro, Waters, Milford, MA, USA) equipped with an electrospray ion source.

### 4.4. Chromatographic Conditions

The UPLC separation was performed using an ACQUITY UPLC BEH Amide 1.7 μm, 2.1 × 50 mm column (Waters Corporation, Milford, MA, USA) operating at a flow rate of 200 μL/min, and eluted with a 4 min linear gradient from 90 to 40% acetonitrile in water (2 mM ammonium formate). The oven temperature was set at 40 °C. The injection volume was 5 μL, and the total analysis time, including 1 min for equilibration of column, was 5 min. 

### 4.5. Mass Spectrometer Conditions

The ESI source operates in negative mode, with a capillary voltage of 2.0 kV and a desolvation temperature of 300 °C. The source of the gas was set as follows: Desolvation at 200 L/h and cone at 0 L/h. The collision cell pressure was 3.50 × 10^−3^ mbar.

The cone voltage and collision energy settings were established individually for each compound for SRM detection. The conditions (Table 2) for the detection of lactulose, mannitol and their internal standards were obtained by direct infusion of a standard solution (1 μg/mL) in line with the HPLC at initial mobile phase conditions. 

### 4.6. Method Validation

To validate the method the following parameters were assessed: linearity, limit of detection (LOD), limit of quantification (LOQ), imprecision, accuracy, recovery and matrix effect. 

The linearity was evaluated by the regression analysis of standards over the concentration range of the calibration curve. LOD and LOQ were evaluated by measuring, in triplicate, serially diluted solutions of lactulose and mannitol from the stock solutions. The LOD was defined as the lowest concentration which gives a signal three times higher than the noise. The LOQ was defined as the lowest concentration that could be measured with a coefficient of variation (CV) < 20% and accuracy between 80 and 120%. The imprecision of the method was evaluated using a urine pool (with lactulose and mannitol not detectable, hereunder named QC0), spiked with three different amounts of sugars (5, 50, 100 µg/mL for lactulose and 20, 100 and 600 µg/mL for mannitol, see Table 1). The three samples were also used as quality control samples (QC1; QC2; QC3). For the evaluation of intra-assay imprecision, each QC was prepared according to the protocol and measured eight times in the same analytical run, while the inter-assay imprecision was evaluated by measuring in duplicate the same QC samples for ten consecutive days. The accuracy was evaluated using ten replicates of QC1, QC2 and QC3, was and expressed as bias%: ([determined value/theoretical value] × 100%). For the recovery study, the urine pool (QC0) was divided into two tubes. The first aliquot (sample 1) was added with different amount of lactulose and mannitol (for the concentration values see Table 1) and treated according to the protocol. The second aliquot (sample 2) was treated according to the protocol and then enriched with the same amount of lactulose and mannitol, in order to achieve the same final concentrations in the two aliquots. The samples were then injected into the LC-MS/MS system. The recovery rate was calculated as the average of (Lactulose)_sample 1_/(Lactulose)_sample 2_ and (Mannitol)_sample 1_/(Mannitol)_sample 2_, and expressed as %. 

For the evaluation of matrix effect, the QC0 was treated according to the protocol after being enriched with different amounts of lactulose and mannitol (5, 50, 100 µg/mL for lactulose and 20, 100 and 600 µg/mL for mannitol). The same amount of lactulose and mannitol was added to a solution with the same composition as the mobile phase in order to have the same final concentration as the enriched urine pool. The samples were then injected into LC-MS/MS system. The results were expressed as the ratio of the areas of each analyte obtained in urine and in mobile phase, and expressed as %.

The stability of lactulose and mannitol were assessed by analyzing in triplicate five samples immediately after preparation and after 24 and 48 h stored at 4 °C and −20 °C. The mean concentration at each level should be within 15% of the nominal concentration.

Reference ranges were evaluated on 50 urinary samples from apparently healthy subjects. 

Data acquisition and data analysis (calibration curve and quantitative analysis) were carried out using the mass spectrometer software (MassLynx and TargetLynx, Waters Corporation, Milford, MA, USA).

Statistical analysis was performed using Microsoft Excel 2010 (Microsoft Office 2010, Redmond, WA, USA). 

## Figures and Tables

**Table 1:** Validation parameters.

Analyte	Nominal Concentration (µg/mL)	Imprecision		Accuracy	
		Intra-Assay CV (%)	Inter-Assay CV (%)	Intra-Assay (%)	Inter-Assay (%)
Mannitol	20	1.3	2.9	98.9	96.6
	100	0.7	4.7	101.2	95.9
	600	1.6	1.9	98.8	97.5
Lactulose	5	2.9	4.1	97.6	94.8
	50	2.3	4.6	99.8	95.9
	100	1.0	2.1	97.2	95.6

**Table 2:** Mass spectrometry condition.

Analyte	Precursor Ion Mass (m/z)	Product Ion Mass (m/z)	Transition	Collision (V)	Cone (V)
Mannitol	181.0	88.9	Quantifier	14	52
	181.0	100.9	Qualifier	14	52
Lactulose	341.2	100.9	Quantifier	14	50
	341.2	160.9	Qualifier	6	50
Lactulose IS	353.2	104.9	Internal standard	16	54
Mannitol IS	184.2	88.9	Internal standard	14	50
